# Nano-superhydrophilic and bioactive surface in poor bone environment. Part 1: transition from primary to secondary stability. A controlled clinical trial: Bioactive implant surfaces in poor density bone

**DOI:** 10.1007/s00784-024-05747-7

**Published:** 2024-06-14

**Authors:** Luigi Canullo, Maria Menini, Paolo Pesce, Roberta Iacono, Anton Sculean, Massimo Del Fabbro

**Affiliations:** 1 Department of Surgical Sciences (DISC), University of Genoa, Largo R. Benzi 10, Genoa, 16132 Italy; 2 Department of Periodontology, University of Bern, Bern, Switzerland; 3 Department of Oral and Maxillo-facial Sciences, “Sapienza” University of Rome, Via Caserta 6, Rome, 00161 Italy; 4 Department of Biomedical, Surgical and Dental Sciences, University of Milan, Milan, Italy; 5 Fondazione IRCCS Ca’ Granda Ospedale Maggiore Policlinico, Milan, Italy

**Keywords:** Implant stability, Implant surface, Bioactive surface, Insertion torque value, Clinical trial, Osseointegration

## Abstract

**Objectives:**

Bioactive surfaces were designed to increase the interaction between the surface and the cells. This may speed up the biological stability and loading protocols.

**Materials and methods:**

36 patients with D3-D4 bone density were recruited and allocated into two groups. 30 bioactive (test group) and 30 traditional (control group) surfaced implants were placed. Insertion torque value (Ncm), insertion torque curve integral (cumulative torque, Ncm), torque density (Ncm/sec), implant stability quotient (ISQ) measured at three timepoints (baseline (T0), 30 (T30) and 45 (T45) days after surgery), and marginal bone loss (MBL) at 6 months of loading were assessed.

**Results:**

The mean ISQ and standard deviation at T0, T30, T45 were respectively 74.57 ± 7.85, 74.78 ± 7.31, 74.97 ± 6.34 in test group, and 77.12 ± 5.83, 73.33 ± 6.13, 73.44 ± 7.89 in control group, respectively. Data analysis showed significant differences between groups in ΔISQ at T0-T30 (*p* = 0.005) and T30-T45 (*p* = 0.012). Control group showed a significant decrease in ISQ at T30 (*p* = 0.01) and T45 (*p* = 0.03) compared to baseline, while no significant change was observed in test group. Due to the stability of the ISQ value ≥ 70, 26 test group and 23 control group implants were functionally loaded after 45 days. Conversely, due to the ISQ < 70 at T45, four test group implants and one control group implant were loaded after 90 days, and 6 control group implants were loaded after 180 days. Neither insertion torque nor ISQ at baseline were correlated with bone density (in Hounsfield units). There was no significant correlation between cumulative torque and ISQ at baseline. There was a significant positive slope in the correlation between torque density and ISQ at baseline, more accentuated in D3 than D4. This correlation remained significant for the test group in D3 bone at day 30 and 45 (*p* < 0.01 in both time frames), but not in D4 bone, and it was not significant in CG.

**Conclusions:**

The bioactive surface showed better behavior in terms of implant stability in D3-D4 bone quality in the early stages of bone healing. Clinical relevance This study demonstrated that the transition from primary to secondary stability is improved using bioactive surface, especially in cases of poor bone environment (D3/D4 bone).

## Introduction

Prosthetic rehabilitation using implants is the first-choice treatment for the edentulous area in order to fully satisfy the patients’ demands in terms of good aesthetic results and functional results in a short period of time and with long-term stability [1].

The growing and high expectations of patients for implant-prosthetic rehabilitation are constantly driving the search for highly performing materials capable of optimizing the osseointegration process and accelerating the transition from primary stability (also known as mechanical stability) to secondary stability (also known as biologic stability ) which is the result of bone turnover that occurs at the implant/bone interface and causes bone resorption and new bone formation (woven bone) [2, 3]. According to some studies in which implant stability was evaluated by resonance frequency analysis (RFA), the transition period from mechanical to biological stability occurs approximately 3–4 weeks after implant insertion, when a decrease in implant stability quotients (ISQ) value is recorded [4–6].

According to Brånemark, osseointegration is a direct structural and functional connection between the implant surface and the host bone. Osseointegration of endosseous implants is achieved by early implant fixation and bone healing [7, 8]. It is possible to think of an adequately osseointegrated implant, which is the final goal of the treatment plan, as the result of a complex mathematical reaction in which each variable has its weight. In this clinical context, the identified variables are the host bone environment and the patient characteristics (including age, gender, and systemic disease), the surgical procedure and the loading protocol, the implant material, design, and surface, among others [9–14].

Especially, when the quality of bone is poor, D3-D4, undersized osteotomy resulted in better BIC values [15]. In fact, additional human randomized controlled studies clarified that osteotomy preparation technique with osteotomes and tapered implant microgeometry resulted in a better implant performance [16, 17].

On the other hand, clinical research has long concentrated on implant surface characteristics, resulting in a substantial volume of studies that explore the clinical implications arising from modifications to implant topography. The macro, micro, and nanoscopic characteristics of the surface of titanium dental implants, by modifying the bone-implant interface and increasing the bone-implant contact (BIC) are able to influence the biological responses that occur in the host from the moment the implant is placed [18].

In particular, the importance of implant surface characteristics is due to the fact that the surface comes into contact with the tissues immediately after implant placement. Macroscopic roughness results in greater BIC and higher resistance to torque removal [19]. Micro-rough implants have proven superior to smooth surface implants and their surface can influence and enhance protein absorption, cell-surface interaction, cell behavior, and cell attachment [20].

Several implant surface treatments have been proposed to modify the roughness of the surface with the aim of making the titanium implant surface osteophilic: titanium plasma-spraying, grit-blasting with hard ceramic particles, anodization, calcium phosphate coatings, and acid etching.

Among these, sandblasted, large grit, acid-etched implant surfaces (SLA) and its chemically modified version storage in saline solution have been the most investigated. Ferguson showed that the removal torque values of the modSLA-surfaced implants were 8–21% higher than those of the SLA implants (*p* = 0.003) [21].

The storage model of SLActive makes it chemically active, with free surface energy, and guarantees strong hydrophilicity compared to non-active surface SLA.

While the initial results are encouraging, reviews have found no substantial differences between SLA and SLA active implants [22]. In terms of implant survival, Sivaswamy demonstrated that in the comparison of SLA versus SLActive, the former showed higher survival rates [23].

New methods have been examined to increase the hydrophilicity of the surface by progressively reducing the contact angle from 138, which is typical of unwettable and hydrophobic surfaces to 0 degrees of superhydrophilic surface.

The aim of the present controlled clinical trial was to evaluate a new implant surface with a bioactive surface which presents sandblasted and acid-etched surface with dry bioactive technology to favor bone healing in a shorter time, and to improve the osseointegration in bone even of poor quality, and to modify the loading protocols.

## Materials and methods

### Study design

A 6-months, single-center, controlled clinical trial was conducted to evaluate the stability curve in post-extractive sites with poor bone quality (D3 and D4) by comparing implant-prosthetic rehabilitation with two different implant surfaces.

Two groups were outlined: test group (TG) or NINA group - receiving bioactive surfaced implants (MultiNeO NH CS, Alpha-Bio Tec, Israel) and control group (CG) or NEO group - receiving traditional moderately rough surfaced implants (MultiNeO CS, Alpha-Bio Tec, Israel). Both implant surfaces were produced by the same manufacturer (Alpha-Bio Tec).

The same surgical procedures were performed in both groups by a single expert clinician (L.C.).

The study protocol was approved by Lazio 1 Ethics Committee (Prot.430/CE Lazio) and was registered within a clinical trials database (www.clinicaltrials.gov) with the registration number NCT 05495867.

Signed informed consent was obtained from all the participants included in the study before the surgery. The study was carried out according to the principles outlined in the Declaration of Helsinki of 1975, as revised in 2013.

### Patient selection

From September 2022 until December 2022, consecutive adult participants in good systemic health, who required implant-prosthetic rehabilitation and presented D3 and D4 bone density, were enrolled at a private dental clinic (Rome, Italy).

Recruitment was performed following specific inclusion/exclusion criteria.

Inclusion criteria were: D3- D4 bone densities; type 1–2 post-extraction healed sites, adult aged 30–80 years; subjects ASA 1 and ASA 2; healthy periodontal conditions (treated periodontitis, PI < 25%, BoP < 25%); patients willing to sign an informed consent and participate in a clinical study.

Exclusion criteria were: absence of type 1–2 bone sites, immediate post-extraction sites, ASA 3 or 4; untreated Periodontitis; any sites where an implant already failed; allergy declared to one or more medicaments to be used during treatment; pregnancy (confirmed by verbal inquiry); severe smokers (less than 10 cigarettes smoked per day was not considered an exclusion criterion).

### Sample size

The sample size calculation was based on the implant stability quotient (ISQ) means and SD of SLA RN (76.5 ± 6.5) and SLActive (78.8 ± 3.20) groups at 12 weeks recorded in a previous study [6]. The following parameters were used: the smallest expected difference between the means; the standard deviation of the difference between the means; a beta error of 20%, an alpha error of 5%, and an effect size of 0.91, as indicated by a sensitivity analysis. These calculations indicated that each group should include a minimum of 20 implants per group [24].

### Presurgical procedures

For all patients, dental evaluation with intraoral photographs, intraoral scanning, and periapical X-ray of the edentulous area was performed. A CBCT was prescribed to analyze the three-dimensional anatomy of the site and plan the implant treatment. Intraoral scanning and CBCT, were used to digitally plan the implant insertion. The specific software (RealGUIDE 5.0, 3Diemme, Cantù, Italy) allowed to measure the bone density (expressed in HU) along the implant length.

**Fig. 1:**
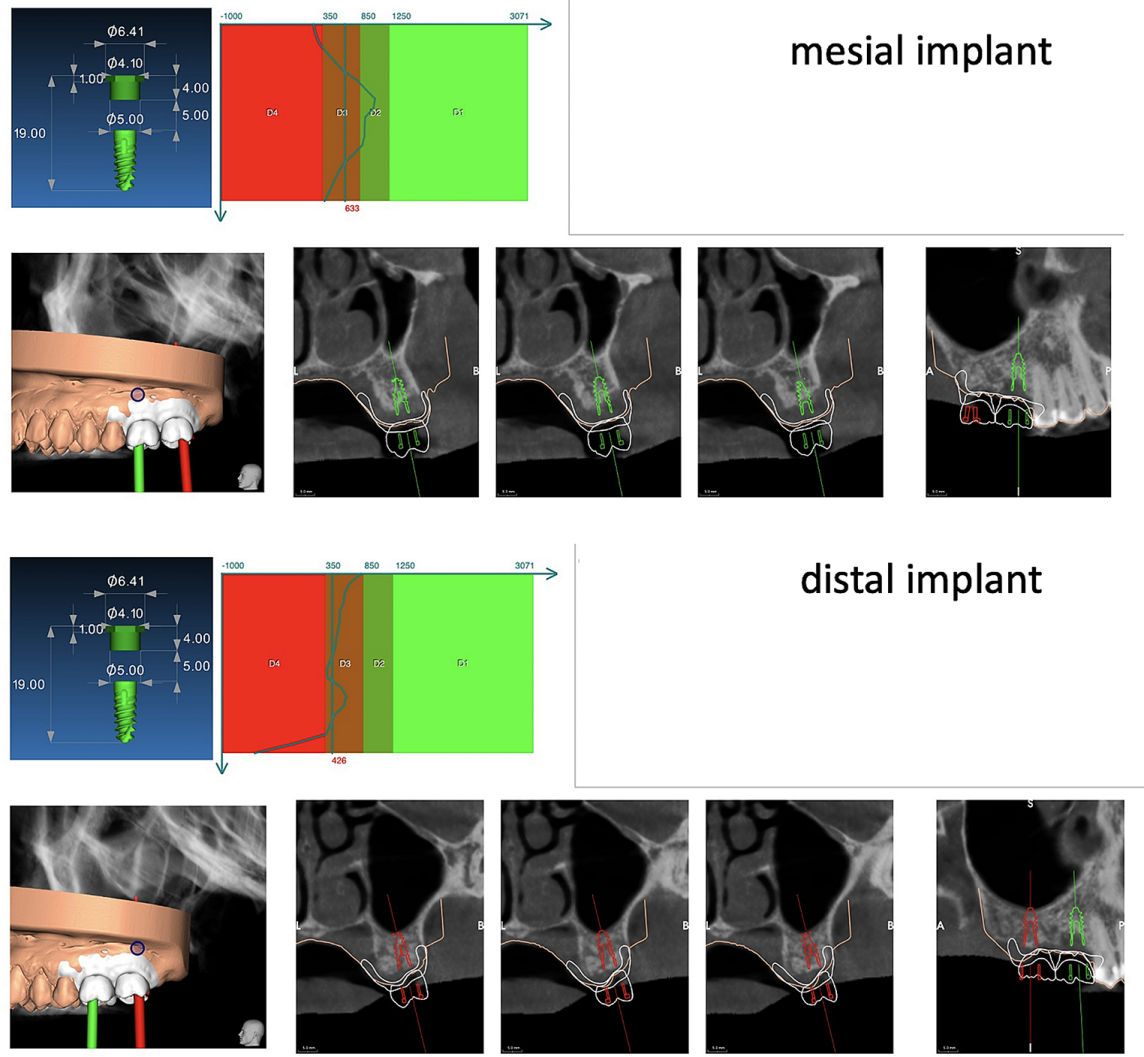
Digital planning of the case. The red/green boxes represent the density in HU measured along the implant length

At the same time, a surgical template was produced for each patient recruited in the study. (Fig. 1)

All patients received professional full mouth tooth cleaning and individual homecare instruction at least two weeks before surgery.

Once selected for the study, patients were consequently assigned to TG, (superhydrophilic surfaced implants, MultiNeO NH, Alpha-Bio Tec, Israel) or CG, (moderately rough surfaced implants, MultiNeO CS, Alpha-Bio Tec, Israel) based on the chronological recruitment order. To obtain two homogeneous samples, age, gender, surgical site (anterior or posterior, upper or lower jaw), and clinical characteristics were taken into consideration.

### Surgical procedure

Following local anesthesia (articaine 4% with adrenaline 1:100.000), patients were instructed to rinse with 0.2% chlorhexidine solution for 5 min. A minimally invasive flap was incised using a 15c blade and raised on the buccal surface of the alveolar process. Once the alveolar ridge was exposed, the surgical template was inserted. The implant site was then prepared using drills with a maximum of 350 rpm and irrigated with a sterile NaCl solution. Following implant site preparation, the surgical template was removed and the implant was inserted to correctly measure the implant stability curve and peak. During the implant insertion, the insertion torque curve (ITC) and the peak of this curve, also called the insertion torque value (ITV), were recorded.

W&H device (SA-310 W&H Elcomed implant units, W&H, Burmoos, Austria) was used for implant insertion and ITC and ITV recording.

**Fig. 2:**
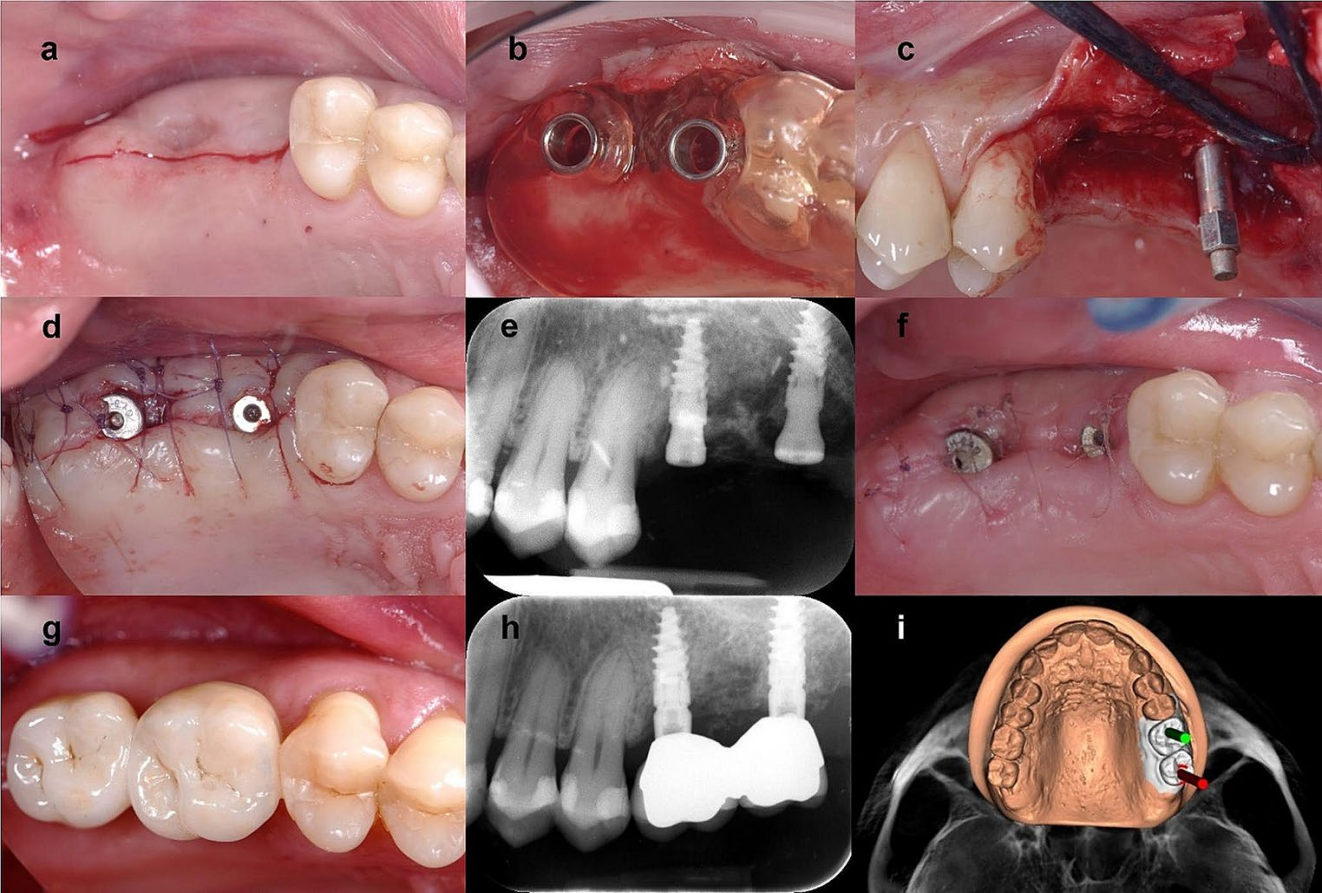
A case of one of 36 patients recruited in this clinical study and treated with two different implant surfaces. The implant in position 26 was MultiNeO CS (control group), while the implant in position 27 was MultiNeO NH CS (test group). (**a**) a crestal incision was made with a 15c scalpel blade. (**b**): A 3D-printed implant surgical guide was used for implant site preparation according to the manufacturer’s protocol. (**c**): The implant stability quotient was measured after implant placement. (**d**): The mucoperiosteal flaps were adapted to the abutments and sutured. (**e**): A periapical X-ray was taken at baseline. (**f**): at 15 days sutures were removed after postoperatively clinical assessment. (**g**): Due ISQ value, the definitive restoration was placed at t45 (**h**) A periapical X-ray was taken at T45.(**i**) Virtual implant planning

At this point a sterile smart peg was screwed into the dental implant and implant stability was checked using Osstell^®^ Mentor device (W&H, Burmoos, Austria). Finally, a healing abutment was inserted and sutures 6.0 was used for flap fixation. A periapical x-ray was performed to assess the post-operative implant placement using Rinn x-ray holder device and the parallel long-cone technique. (Fig. 2a-e).

### Postsurgical procedure

Systemic antibiotics (amoxicillin/clavulanic acid 875 mg/125 mg) were prescribed the night prior to surgery and 3 times daily for 4 days after surgery 22. Analgesics (ibuprofen 600 mg) were prescribed to be taken only as needed.

All the patients were instructed to rinse with 0,12% chlorhexidine and 0,05% cetylpyridinium chloride twice a day for 15 days starting on the day after surgery [25]. They were instructed to avoid brushing and chewing at the surgical site to prevent mechanical trauma. Moreover, patients were instructed to apply cold packs over the treated area to minimize the inflammatory response and decrease post-operative swelling.

At 15 days after surgery, the suture was removed after a clinical examination. (Fig. 2f)

All patients were included in an individual maintenance program with professional mechanical tooth cleaning every 3 or 4 months, depending on the case.

### Prosthetic procedure

30 days after implant placement, the healing abutment was removed, and implant stability was assessed using a smart peg and Osstell^®^ Mentor device (W&H, Burmoos, Austria). A digital impression was taken and sent to the dental lab for the fabrication of the prosthetic restoration. Implant stability measurements were repeated 45 days after implant placement.

Due to the stable ISQ curve, NINA implants and NEO implants were functionally loaded after 45 or 90, or 180 days and a periapical x-ray was performed. (Fig. 2g-h)

The loading phase was allowed before the traditional osteointegration timing (90–180 days) only if, at 45 days, the ISQ curve was basically flat.

In case, the curve resulted slopped, the traditional timing was maintained.

### Clinical and radiographic measures

Only surgical sites with D3 and D4 bone density were recruited. These densities are expressed numerically with the Hounsfield scale, D4 < 350 HU; D3 350–850 HU. Bone densities were assessed from preoperative CBCT in Hounsfield units (HU) using RealGUIDE 5.0 software (3Diemme, Cantù, Italy).

Implant stability, the main outcome of the study, was checked at three different time points: Baseline (immediately after implant insertion) (T0) and at 30 (T1) and 45 (T2) days after the surgery. A smart peg was screwed into the implant connection and Osstell^®^ Mentor (W&H, Burmoos, Austria) device was used to perform resonance frequency analysis to determine ISQ. Two measurements were taken at each time point, one bucco-palatally from the buccal side of the implant and one mesiodistally from the mesial side. The ISQ value is the mean value of these two measurements.

The integral of the Torque insertion curve (cumulative torque, Ncm) was calculated by the sum of each single torque value from the start of insertion until the achievement of the peak insertion torque. The torque density (Ncm/sec) was estimated by dividing the cumulative torque by the total time (seconds) required to achieve the peak torque at insertion.

Periapical x-rays, taken with Rinn x-ray holders, were performed at baseline and 6 months after implant placement. Radiographs were used to calculate early marginal bone loss (MBL) comparing the mesial and distal implant margin bone changes.

### Statistical methods

The descriptive statistics were performed using mean data and standard deviations for the quantitative variables normally distributed, median and 95% confidence intervals for quantitative variables not normally distributed, and frequencies for qualitative variables. The mixed effects linear regression model was used to evaluate the effect of predictive factors on the outcome variable. The latter was ISQ at baseline, 30, and 45 days, and MBL. Predictive factors were age, bone density, insertion torque, cumulative torque, and torque density (for MBL, also ISQ at baseline was considered). Simple regression was also performed to determine the correlation between torque density/cumulative torque and ISQ, between torque density/cumulative torque and insertion torque (Ncm), and between ISQ changes at 30 and 45 days and MBL. Both overall data and data obtained with the two implant systems used (MultiNeo CS and MultiNeo NH CS) were considered. Comparisons between the two groups of implants were made using unpaired Student’s t-test for quantitative variables and Pearson’s chi square or Fisher’s exact test, as appropriate, for qualitative scores. Implant-based analyses were performed. Adjusted prediction analysis was performed to estimate the trend of ISQ at baseline, 30 and 45 days, according to cumulative torque and torque density. The data were sorted using Microsoft Excel 2019, and statistical analysis was performed using GraphPad Prism 5.03 (GraphPad Software, Inc., La Jolla, CA, USA), and STATA version 17 (StataCorp, College Station, TX, USA). The significance level was considered at *P* = 0.05.

## Results

A total of 30 MultiNeO CS (Control group- CG) and 30 MultiNeO NH CS (Test group- TG) implants were inserted in 36 patients. Table 1 summarizes the main characteristics of the patients for both treatment groups. Tables 2 and 3 summarize the main clinical and radiographic results of the two groups of implants.

**Table 1:** Demographic parameters

Patient characteristic	Control group *N* = 30	Test group *N* = 30	*P*-value (t-test)
Age, years	61.63 ± 9.61	59.06 ± 11.44	0.35
Gender (Male/Female)	14 /16	11/19	0.37*
ASA (1/2)	18 /12	23 /7	0.14*
Bone density (HU)	480.47 ± 177.09	449.90 ± 195.58	0.53
Bone density (D3/D4)	22/8	18/12	0.32*

*Pearson’s chi square

**Table 2:** Clinical and radiographical measurements

Clinical and radiographical measurements	Control group	Test group	*P*-value (t-test)	*P*-value (non-param.)
Peak insertion torque (Ncm)	33.07 ± 9.22	32.19 ± 11.70	0.75	
Cumulative torque (Ncm)	1361.4 ± 562.9	1396.6 ± 570.1	0.81	
Seconds to peak	9.66 ± 2.63	11.11 ± 5.56	0.20	
Torque density (Ncm/sec)	139.53 ± 37.01	131.40 ± 46.63	0.46	
MBL 6-month (mm) median (95% CI)	0.30 ± 0.49 0.00 (0.12, 0.48)	0.31 ± 0.35 0.20 (0.18, 0.44)		0.21***
MBL 6 m D3 (mm) median (95% CI)	0.37 ± 0.56 0.05 (0.10, 0.64)	0.37 ± 0.040 0.30 (0.18, 0.56)		0.31***
MBL 6 m D4 (mm) median (95% CI)	0.17 ± 0.26 0.00 (0.00, 0.34)	0.22 ± 0.26 0.125 (0.05, 0.38)		0.46***
P-value D3 vs. D4‡	0.43	0.21		

***Mann Whitney test; ‡=Wilcoxon matched-pairs test

**Table 3:** Implant stability at the different time points and the ISQ behaviour in the two treatment groups

Implant stability quotient	Control group	Test group	*P*-value (t-test)	*P*-value (non-param.)
Mean ISQ T0	77.12 ± 5.83	74.57 ± 7.85	0.16	0.36***
Mean ISQ T30	73.33 ± 6.13	74.78 ± 7.31	0.41	0.52***
Mean ISQ T45	73.44 ± 7.89	74.97 ± 6.34	0.41	0.71***
Δ T30-T0	-3.78 ± 7.50	0.21 ± 3.97	**0.005**	0.0375***
Δ T45-T0	-3.68 ± 8.82	0.40 ± 4.36	**0.012**	0.0396***
P-value Δ T30 vs. T0 (paired)	**0.017‡**	0.52‡		
P-value Δ T45 vs. T0 (paired)	**0.017‡**	0.72‡		
ISQ D3 (T0)	77.66 ± 6.17	74.78 ± 8.53	0.22	0.18***
ISQ D3 (T30)	72.73 ± 6.86	75.17 ± 8.44	0.32	0.23***
ISQ D3 (T45)	73.95 ± 8.93	75.14 ± 7.56	0.66	0.85***
Δ D3 T30-T0	-4.93 ± 8.24	0.39 ± 3.72	**0.016**	0.0374***
Δ D3 T45-T0	-3.70 ± 9.78	0.36 ± 3.78	0.10	0.15***
P-value D3 T30 vs. T0 (paired)	**0.014‡**	0.34‡		
P-value D3 T45 vs. T0 (paired)	**0.047‡**	0.60‡		
ISQ D4 (T0)	75.63 ± 4.82	74.25 ± 7.06	0.64	0.97***
ISQ D4 (T30)	75.00 ± 3.18	74.19 ± 5.49	0.71	0.67***
ISQ D4 (T45)	72.03 ± 3.96	74.71 ± 4.17	0.17	0.15***
Δ D4 T30-T0	-0.63 ± 3.73	-0.06 ± 4.47	0.77	0.67***
Δ D4 T45-T0	-3.59 ± 5.93	0.46 ± 5.30	0.13	0.16***
P-value D4 T30 vs. T0 (paired)	0.61‡	0.64‡		
P-value D4 T45 vs. T0 (paired)	0.195‡	0.61‡		

***Mann Whitney test; ‡=Wilcoxon matched-pairs test

Despite a very similar peak insertion torque, CG achieved a slightly (but not significantly) higher mean ISQ than TG at placement. Over time, the ISQ in CG decreased significantly, while it remained stable in TG, the changes (Δ respect to baseline) being significantly different at 30 (*p* = 0.01) and 45 (*p* = 0.03) days. The pattern was similar in D3 and D4 bone; in CG, the decrease was significant in D3 but not in D4, and in TG, ISQ change was not significant in both D3 and D4. No significant differences between groups were observed in MBL.

**Fig. 3:**
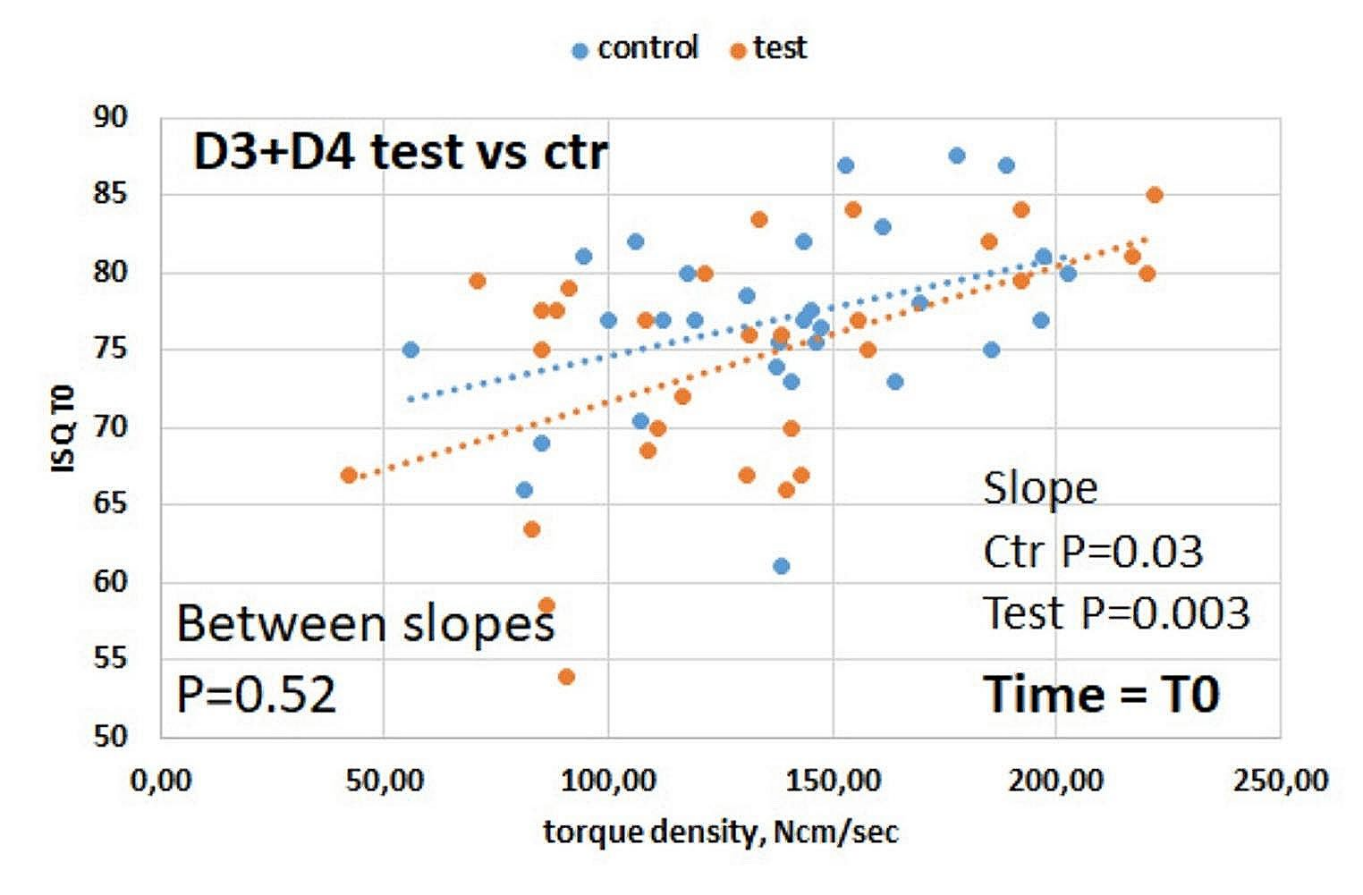
Correlation between torque density and ISQ at baseline. A significant correlation was observed in both groups, indicating that implant stability may be affected by the torque density

**Fig. 4:**
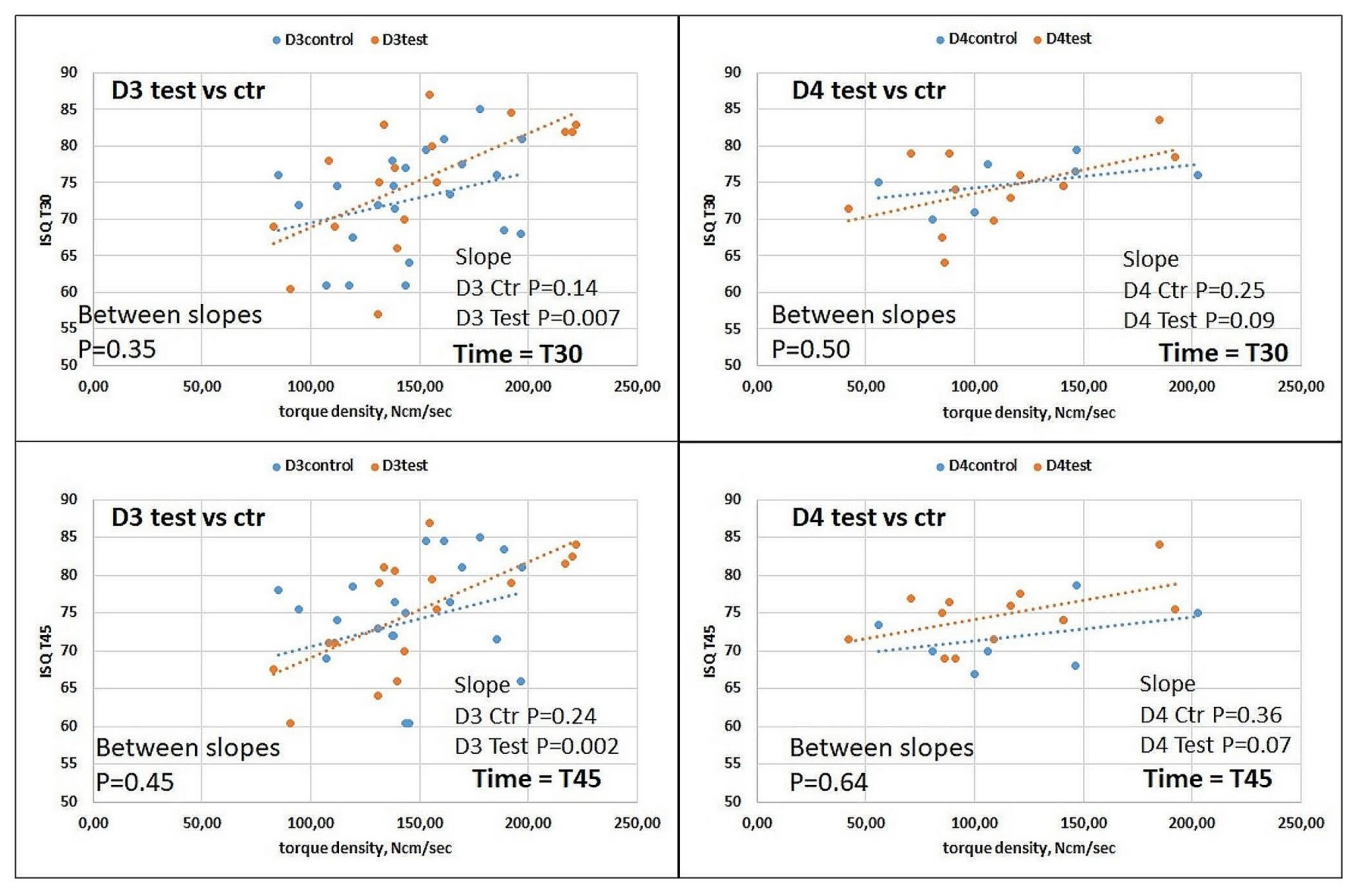
Correlation between torque density and ISQ at 30 days (upper panels) and 45 days (lower panel). A significant correlation was observed in test group in D3 (left panels), but not in D4 (right panels), suggesting that superhydrophilic surfaces may be affected by the torque density, especially when they are positioned in D3 bone, as opposed to control surfaces

Neither insertion torque (Ncm) nor ISQ at baseline were correlated with bone density (HU). There was no significant correlation between cumulative torque (integral of the torque curve) and ISQ at baseline, suggesting that implant-bone interface stability at placement is relatively independent of the pattern required to achieve the final insertion torque. There was a significant positive slope in the correlation between torque density (Ncm/sec) and ISQ at baseline (Fig. 3), more accentuated in D3 than D4. This suggests that implant stability at placement may depend on the pattern required to achieve the final insertion torque. This correlation remained significant for the test group in D3 bone at day 30 and 45 (*p* < 0.01 in both time frames), but not in D4 bone (0.05 < *p* < 0.10 in both time frames), and it was not significant in CG (Fig. 4).

**Fig. 5:**
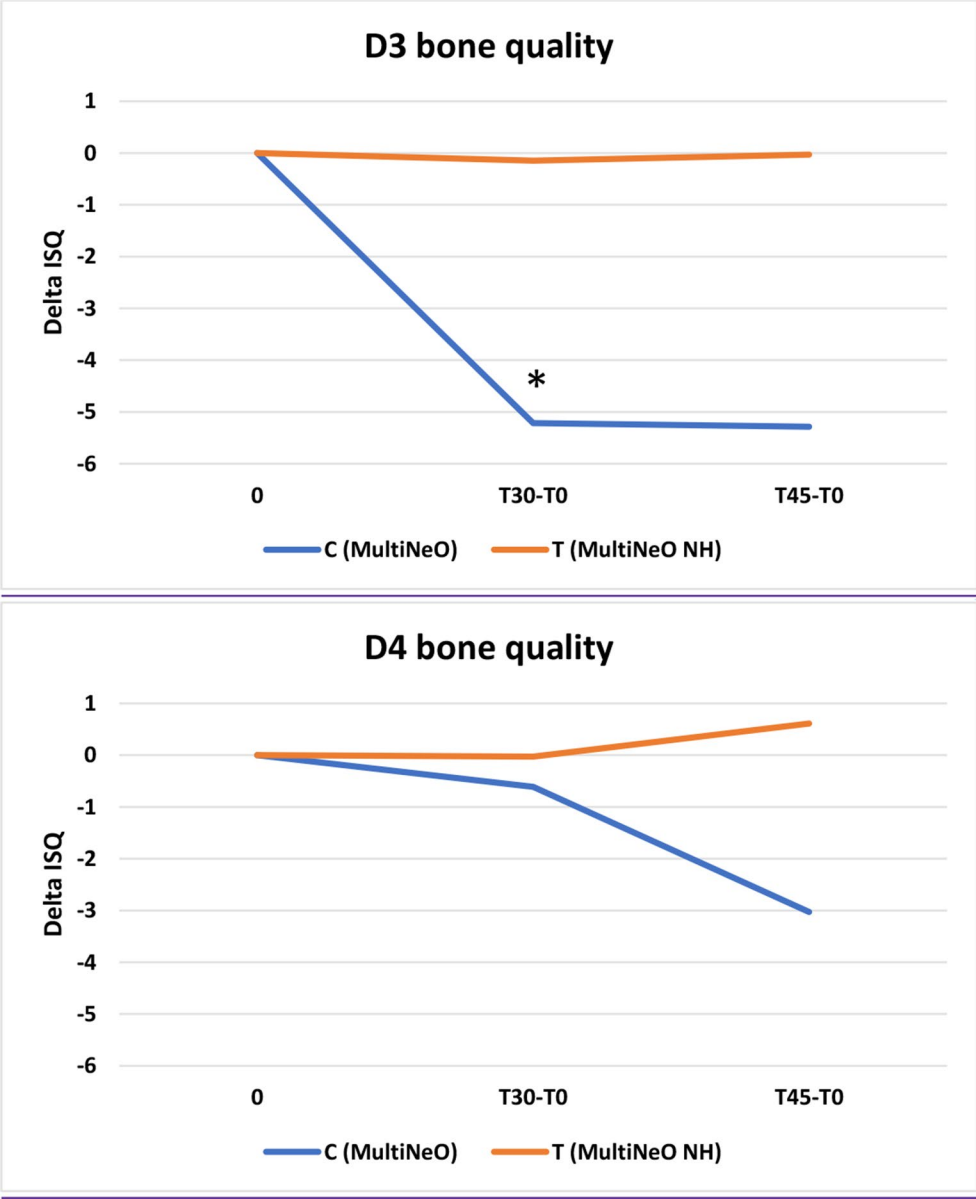
Changes in ISQ value at T30 and T45 in D3 bone (upper panel) and D4 bone (lower panel). In the D3 bone, implants with superhydrophilic surface showed a small variation in ISQ, while control implants displayed a significant drop at 30 days. Non-significant changes in ISQ were recorded for implants placed in D4 bone

Figure 5 shows the changes in ISQ value at T30 and T45 in D3 bone (upper panel) and D4 bone (bottom panel). In the D3 bone, implants with superhydrophilic surface showed a very small variation in ISQ, while control implants displayed a significant drop at 30 days. Non-significant changes in ISQ were recorded for implants placed in D4 bone (bottom panel).

**Fig. 6:**
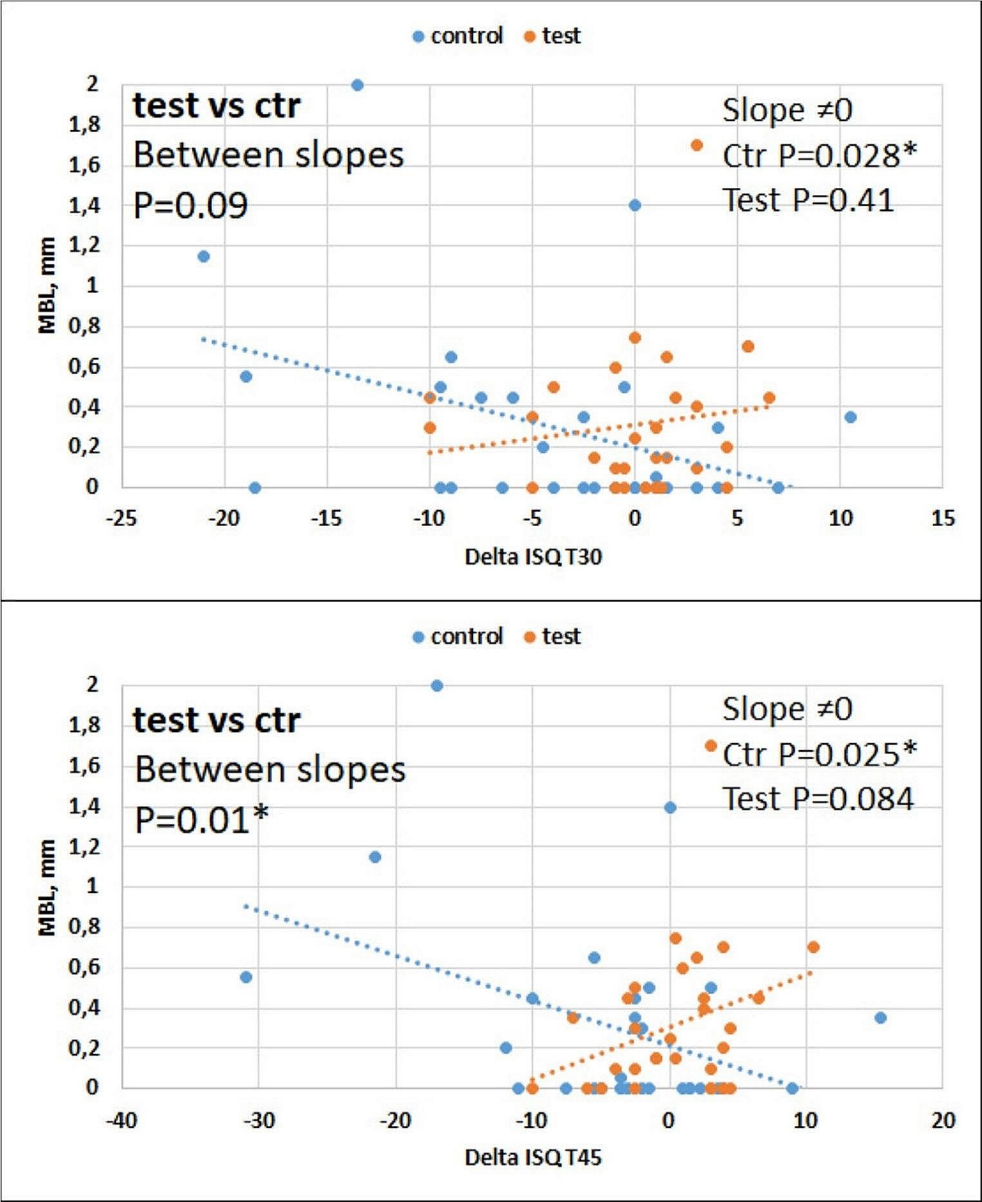
Correlation between MBL and ISQ change at T30 (upper panel) and T45 (lower panel). Implants with superhydrophilic surface showed a small variation in ISQ, while control implants displayed a significant negative slope, indicating that the greater the loss of primary stability, the higher the MBL, as opposed to the control group in which the slope was not significant. The difference between groups was significant at T45

MBL was significantly correlated to delta ISQ T30 and T45 in CG, with a negative slope (the greater the ISQ drop, the higher the MBL), and showed an opposite trend in TG, although not significant (Fig. 6). Implant stability might be affected by the change in MBL over time, which could be a consequence of remodelling around the implants. This remodelling appears to be lower in TG implants, which showed a minimal change of ISQ The difference between slopes of the test and control group for the correlation between ISQ change and MBL became significant at T45 (bottom panel of Fig. 6). It can be noticed that the range of variation of both ISQ change and MBL was greater for the control than the test group implants. When considering D3 and D4 bone separately, the same trend was observed but significance was not always achieved, probably due to the small sample size in the D3 and D4 subgroups.

No intraoperative and postoperative complications were observed. No implant failure occurred. The stable ISQ curve was considered a prerequisite for implant loading, and based on this curve, 26 TG implants and 23 CG implants were functionally loaded after 45 days. Conversely, four TG and one CG implants, two ISQ values at T45 < 70, were functionally loaded after 90 days. Six CG implants were loaded after 180 days.

The mixed effects linear regression analysis showed that only torque density had a significant effect on ISQ at 30 and 45 days, while no variable had an effect on MBL (Table 4A-D).

**Table 4:** Mixed-effect linear regression analysis for ISQ T0 (A), ISQ T30 (B), ISQ T45 (C), and MBL at 6 months (D). A

Factor	Coefficient	Std.Err.	Z	P_value	95% CI
**A**					
Age	0.0406	0.0819	0.50	0.620	-0.120, 0.201
Bone density (HU)	0.0004	0.0045	0.10	0.924	-0.008, 0.009
Insertion Torque	0.2379	0.1298	1.83	0.037	-0.016, 0.492
Cumulative torque	-0.0028	0.0017	1.63	0.102	-0.006, 0.0006
Torque density	0.0540	0.030	1.79	0.074	-0.005, 0.113
**B**					
Age	-0.003	0.084	-0.04	0.971	-0.168, 0.162
Bone density (HU)	0.0008	0.0047	0.17	0.864	-0.008, 0.01
Insertion Torque	-0.0265	0.1334	-0.20	0.843	-0.288, 0.235
Cumulative torque	-0.003	0.0018	-1.69	0.091	-0.007, 0.0005
Torque density	0.0935	0.0308	3.03	0.002	0.033, 0.154
**C**					
Age	-0.1257	0.0887	-1.42	0.156	-0.299, 0.048
Bone density (HU)	-0.0009	0.0049	-0.18	0.859	-0.01, 0.009
Insertion Torque	-0.0042	0.1407	-0.03	0.976	-0.28, 0.272
Cumulative torque	-0.0022	0.0019	-1.15	0.252	-0.006, 0.002
Torque density	0.085	0.0325	2.61	0.009	0.212, 0.149
**D**					
Age	0.0002	0.0055	0.04	0.971	-0.011, 0.011
Bone density (HU)	0.0006	0.0003	1.85	0.064	-0.000, 0.001
Insertion Torque	-0.0057	0.009	-0.63	0.526	-0.023, 0.012
Cumulative torque	0.0001	0.0001	1.11	0.268	-0.0001, 0.0004
Torque density	0.0008	0.0021	0.37	0.709	-0.003, 0.005
ISQ T0	-0.0079	0.0087	-0.90	0.367	-0.025, 0.009

## Discussion

The aim of this clinical study was to evaluate, through the stability curve and the ISQ value, whether a superhydrophilic and bioactive implant surface may represent a key factor to improve and accelerate the osseointegration process and favor early loading protocols even when conditions are less favorable, such as in the case of bone type D3 or, particularly, D4.

The design of this study is similar to that of previous studies conducted over the last 15 years, which have compared various bioactive surfaces with traditional ones.

In fact, recent systematic reviews have shown no significant difference in terms of stability between implants with super-hydrophilic surfaces and implants with traditional surfaces [26, 27].

One possible reason for this absence of difference may be associated with the heterogeneity of the sample size, primarily comprising implants inserted in the posterior mandible. The anatomy of this region may indeed feature a substantial cortical bone area that is susceptible to alterations, potentially causing the implant stability curve to flatten and leading to a combined measure of primary and secondary stability.

To avoid this bias, the present study was carried out also considering the bone environment and the different responses of the host where the implant is inserted. This is the reason why only sites with poor densities (< 850 HU) were included. In fact, Chrcanovic and colleagues (2017) have highlighted that sites with poor bone quality and lack of bone volume can substantially influence implant failure rates [9].

On the other hand, in vitro studies have demonstrated that bioactive surfaces allow a significant quantitative and qualitative advantage in terms of cell adhesion and stratification [28]. However, this phenomenon seems to have a short effect due to the so-called “saturation effect”. Indeed, statistically significant differences exhibit a strong inverse correlation with cell concentration: the higher the concentration, the shorter the period of significant difference. Simultaneously, a direct correlation is observed between the surface area and the time required for the saturation effect.

In the present study, implants with a superhydrophilic and bioactive surface were placed in the TG patients. While the NINA group showed a constant increase in ISQ values at T30 e T45 (75.15 ± 7.39, 75.31 ± 6.49) compared to the baseline (74.72 ± 7.70), in the NEO group, a decrease in stability was noted at T30 (73.26 ± 6.04) and T45 (73.23 ± 7.85) compared to the baseline (77.11 ± 5.73). Implant stability was statistically significantly higher at T30 (*p* = 0.005) and T45 (*p* = 0.012) with the bioactive surfaces. A significant drop in ISQ was observed in the implants placed in D3 bone in the NEO group (*n* = 22), as opposed to implants in D3 bone in the NINA group (*n* = 18). The between-group difference in ISQ behavior cannot be justified only by the slight difference in proportion of implants placed in D3/D4 bone in the two groups but is surface-related. These results appear to suggest that a more rapid transition from mechanical to biological stability, and therefore faster formation of woven bone, occurred in TG. The TG data are in contrast with other studies described in the literature. Khandelwal in the study comparing SLA with chemically modified SLA showed that ISQ levels decreased after implant placement and the minimum in implant stability was achieved at 3–4 weeks after placement in both treatment groups [25]. Barewal demonstrated that the most critical period for implant stability occurs around week 3 after implant placement for all bone types, especially bone type 4 [29]. Bornstein in contrast to Khhandewal showed that the stability of modSLA at 4 months was (77.91 ± 6.00), showing an increase in stability from baseline (74.33 ± 7.06) [30]. However, both studies lacked sub-analyses of implant site bone quality which could have influenced the stability values.

According to the ISQ trend, despite the critical bone quality, 26 TG and 23 CG implants were functionally restored according to the study protocol at day 45. 6 CG and 0 TG implants were functionally loaded after 180 days.

These results are in accordance with Bornstein et al. who showed a high success rate (96%) in the posterior mandible, loading bioactive implants after 21days. Despite similar results, the differences in loading time between the aforementioned and the present study might be related to bone quality.

These results could be explained by significant differences in wettability between the groups. Superhydrophilic surfaces with a contact angle (CA) of 0 degrees, are completely imbibed by blood and, as already demonstrated in previous in vitro animal and clinical studies, promote protein adsorption, cell adhesion, as well as proliferation and differentiation of human mesenchymal stem cells in osteoblasts, thus promoting bone healing already at the early stages after implant placement [31–34].

Despite a follow-up of only 6 months, all 60 implants were functionally loaded and adequately osseointegrated, with a survival and success rate of 100.0%, confirming the predictability of implant therapy.

In the present study, the use of computer-guided surgery has simplified the surgical procedure and eliminated surgical complications [35, 36]. In fact, none of the implant site preparations deviated substantially from the planning carried out with the software. The same software was used to calculate the bone density using the Hounsfield scale and providing information on the bone quality of the surgical site.

The MBL at 6 months was 0.20 (0.18, 0.44) mm and 0.00 (0.12, 0.48) mm respectively for TG and CG with no statistical significance (*p* = 0.21). The absence of statistically significant differences and a low MBL value can be attributed to the coronal macromorphology (presence of Cutting flutes on the coronal portion) of the two implants used. These cutting flutes are specifically designed for a drastic reduction of crestal stress. However, there is still little evidence of the influence of the implant surface on the MBL [37].

This data should be compared to 0.488 mm (95% CI 0.289–0.687), the MBL found in Sommer’s systematic review for early loading in which the prosthetic restoration is placed more than 2 days but less than 90 days after implant placement. The authors estimate that maximum MBL occurs in the first six months following the insertion of the restoration and that tissue stabilization occurs thereafter. No statistically significant differences are expected between the MBL calculated at 6 and 12 months [38].

When interpreting the current findings, it’s important to consider that the relatively short follow-up period constitutes a significant limitation of this study. Nevertheless, despite this limitation, the study’s well-balanced sample size allows for valuable insights into the clinical significance of super-hydrophilic implants.

Based on these promising short-term results, it seems that titanium implants with a bioactive super-hydrophilic surface could serve as a potential treatment option for challenging bone sites, potentially enabling the reduction of loading protocols. Another challenge will be to conduct a more extensive assessment of the performance of this novel bioactive surface under conditions of both low bone quality and limited bone quantity.

## Conclusion

Within the constraints of this study, the data indicate that bioactive super-hydrophilic surface implants exhibit significantly improved stability in D3-D4 bone quality during the early stages of bone healing when compared to traditional moderately rough surfaces. This distinction suggests a potentially faster transition from mechanical to biological stability. In clinical practice, these findings imply that employing a bioactive super-hydrophilic surface may allow for functional loading as early as 45 days, even in D3-D4 bone conditions. Notably, early loading does not appear to negatively impact marginal bone loss (MBL). Nonetheless, a more extended follow-up is recommended to substantiate these clinical implications.

## Data Availability

Data are available on request to the corresponding author.
