# Correction to: Nano-superhydrophilic and bioactive surface in poor bone environment. Part 1: transition from primary to secondary stability. A controlled clinical trial

**DOI:** 10.1007/s00784-024-05826-9

**Published:** 2024-07-12

**Authors:** Luigi Canullo, Maria Menini, Paolo Pesce, Roberta Iacono, Anton Sculean, Massimo Del Fabbro

**Affiliations:** 1 Department of Surgical Sciences (DISC), University of Genoa, Largo R. Benzi 10, Genoa, 16132 Italy; 2 Department of Periodontology, University of Bern, Bern, Switzerland; 3 Department of Oral and Maxillo-facial Sciences, “Sapienza” University of Rome, Via Caserta 6, Rome, 00161 Italy; 4 Department of Biomedical, Surgical and Dental Sciences, University of Milan, Milan, Italy; 5 Fondazione IRCCS Ca’ Granda Ospedale Maggiore Policlinico, Milan, Italy

**Correction to: Clinical Oral Investigations**

10.1007/s00784-024-05747-7.

The published paper revealed that the names and family names of the author group had been reversed.

The original article has been corrected.

